# Exploring patient preferences regarding the use of combination therapy with endothelin receptor antagonist (ERA) + phosphodiesterase-5 inhibitors (PDE5i)

**DOI:** 10.1016/j.jhlto.2025.100410

**Published:** 2025-10-15

**Authors:** Nicholas A. Kolaitis, Martha Kingman, Melisa Wilson, Gabriela Gomez Rendon, David Lopez, Carly J. Paoli, Mohammad Rahman, Ashley Martin, November McGarvey, Abraham Lee, Sana Mirza, Lana Melendres-Groves

**Affiliations:** a Department of Medicine, Division of Pulmonary and Critical Care Medicine, University of California, San Francisco, San Francisco, California; b University of Texas Southwestern Medical Center, Dallas, Texas; c Advanced Lung Disease, AdventHealth Medical Group, Orlando, Florida; d Johnson & Johnson Innovative Medicine, Titusville, New Jersey; e BluePath Solutions, Los Angeles, California; f Division of Pulmonary and Critical Care Medicine, University of New Mexico, Albuquerque, New Mexico

**Keywords:** pulmonary arterial hypertension, combination therapy, treatment preferences, ERA, PDE5i

## Abstract

**Background:**

To evaluate patient preferences for endothelin receptor antagonist (ERA) + phosphodiesterase-5 inhibitor (PDE5i) combination therapies in managing pulmonary arterial hypertension (PAH), including the potential benefits of a single tablet combination therapy (STCT) in reducing pill burden and improving adherence.

**Methods:**

An online survey was administered to 201 adult patients with PAH residing in the United States. Patients' preferences toward oral PAH therapies, including willingness to use double combination ERA+PDE5i therapy, were assessed using a discrete choice experiment (DCE).

**Results:**

Respondents were predominantly White (86.1%) and female (88.6%), with a median age of 59 years. Most were not employed (56.2%), and 38.3% reported a disability. At the time of the survey, 36.8% were on triple therapy (PDE5i + ERA + prostacyclin), 17.9% on dual therapy (PDE5i + ERA), and the remaining on other or no therapy. Out-of-pocket costs and dosing regimen were the top factors influencing therapy choice in the DCE, with patients preferring lower out-of-pocket costs and simpler dosing regimens. Regarding STCT, 83.1% believed it would reduce pill consumption, 68.7% thought it would decrease time spent managing prescriptions, and 34.8% and 39.3% anticipated improvements in treatment initiation and adherence, respectively.

**Conclusions:**

STCT offers potential benefits in reducing pill burden, improving convenience, and possibly facilitating treatment initiation and adherence. However, cost remains a significant factor influencing patient decision-making in PAH therapy in the United States. Addressing cost concerns may increase the acceptance of ERA + PDE5i therapies and STCT among patients with PAH.

## Background

Pulmonary arterial hypertension (PAH) is a rare, progressive disease characterized by the proliferation and remodeling of the pulmonary vasculature.^1^ This pathological obstruction leads to elevated pulmonary vascular resistance and increased right ventricular afterload. As a result, the heart's capacity to function as a hemodynamic pump is impaired, reducing its ability to deliver sufficient oxygen to the body at the rate required for normal metabolizing tissue.^2^ Symptoms include chest pain, dyspnea, fatigue, and functional impairment, which worsen gradually. In addition to diminishing health-related quality of life, this physiological maladaptation of the heart eventually leads to right ventricular failure, high health care resource utilization, and premature death.^2^, ^3^, ^4^

The US Food and Drug Administration (FDA) has authorized a range of treatments for PAH via 4 distinct pathways. Although upfront combination therapy is considered the standard of care in PAH management due to its superior clinical outcomes, monotherapy remains widely used despite extensive evidence supporting the benefits of combination treatments.^5^, ^6^, ^7^, ^8^ Indeed, the proceedings of the 7th World Symposium on Pulmonary Hypertension recommend that all newly diagnosed Group 1 patients with PAH be started on upfront combination therapy with a phosphodiesterase-5 inhibitor (PDE5i) and an endothelin receptor antagonist (ERA), given the improved observed outcomes.^4^ However, the complexity of PAH treatment regimens poses significant challenges. Patients often require medications with diverse routes of administration. PAH therapies can be oral, inhaled, continuous infusion, or via subcutaneous injection. These medications also require frequent dosing schedules in addition to other concurrent therapies to manage comorbidities. Over 80% of patients with PAH take 5 or more medications, contributing to polypharmacy and reduced adherence.^9^ A separate US claims study indicated that patients with PAH take a mean (standard deviation) of 11.8 (8.2) tablets per day.^10^

Recently, the FDA approved a single tablet combination therapy (STCT), which combines the PDE5i tadalafil and ERA macitentan in a once daily tablet.^11^ In other fields of medicine, STCTs are considered an effective strategy for enhancing the accessibility and adherence to treatment.^12^ STCT can also lead to better disease control in other cardiovascular diseases, such as hypertension, dyslipidemia, and diabetes.^13^, ^14^, ^15^, ^16^, ^17^, ^18^, ^19^

Although STCT in PAH is now available, the current scientific literature is largely missing the patients’ perspectives in the context of these emerging treatment options. We sought to identify the key drivers guiding the patients’ choice of PDE5i + ERA combination therapy, and to assess their receptivity to utilize a STCT formulation.

## Methods

### Study design

An online survey was administered to a US sample of patients with PAH to assess their beliefs and attitudes around the adoption of double combination ERA + PDE5i therapy for managing their PAH. The survey evaluated patients’ (1) demographics and health characteristics, (2) opinions regarding STCT, and (3) treatment attributes driving their decision to select ERA + PDE5i using a discrete choice experiment (DCE). All content was designed, evaluated, and pilot tested by a panel of 3 independent PAH practitioners to ensure content validity and real-world relevance. The study was reviewed and granted exemption by the Western Institutional Review Board-Copernicus Group. Participant recruitment and outreach activities were managed by the Pulmonary Hypertension Association Registry (PHAR).

### Participant population

Participants were sourced from PHAR, the largest active longitudinal registry in the United States that tracks patients with PAH who are new to care at a designated Pulmonary Hypertension Association Care Center.^20^ Registrants were emailed by PHAR staff alerting them to the availability of an optional research opportunity, and the first 201 who met eligibility criteria and completed the online survey were included in the analysis. No identifiable data were collected as part of the survey and the survey results were not linked back to the PHAR dataset.

Eligible participants were aged 18+, diagnosed with PAH, and had used at least 1 of the following PAH medications for 3 continuous months within the past year: Ambrisentan, Bosentan, Macitentan, Sildenafil, Tadalafil, Selexipag, Riociguat, Treprostinil, Epoprostenol, or Iloprost. Participants were ineligible if they self-reported any diagnosis of chronic thromboembolic pulmonary hypertension, interstitial lung disease, and/or diastolic heart failure. All participants were required to be able to speak, read, and understand English. Data were collected between August and September 2023.

## Data collection

### Participant demographics and health characteristics

Participant demographics (e.g., age, sex) and health characteristics (e.g., comorbidities, current treatments) were assessed via self-reported questionnaire items.

### Treatment factors influencing ERA + PDE5i adoption

A DCE was conducted to evaluate which treatment factors were most important in determining patients’ willingness to adopt guideline–recommended double combination therapy. Each respondent completed a series of choice tasks in which they reviewed 3 profiles of hypothetical PAH treatments and selected the profile they found most appealing (Supplement S0.1). Each treatment profile presented a randomized combination of 7 different attributes, each with its own set of response options (“attribute levels”): (1) out-of-pocket costs, (2) dosing regimen, (3) percent of patients who discontinued medication due to side effects, (4) number of prior authorizations required, (5) number of pharmacies needed to visit, (6) number of dose increases (titration), and (7) patient support program (Supplement S0.2). One option/level for each of the 7 attributes was shown per profile, and the combinations of attributes were automatically selected based on an adaptive conjoint methodology.^21^ No 2 versions of the DCE were the same across respondents, minimizing the opportunity for bias.^22^ Key outcomes of the DCE included *preference weights* (or “part-worth utilities”), which were used to evaluate *within*-attribute treatment patterns, and *relative importance estimates*, which were used to evaluate *between*-attribute treatment patterns.

### Perceptions of STCT for ERA + PDE5i adoption

After completion of the DCE, each respondent was shown 4 blinded ERA + PDE5i therapies and asked to select the 1 therapy they found most appealing (Supplement S0.3). Three of the blinded regimens were modeled off FDA-approved loose-tablet double combination therapies of ERA and PDE5i (macitentan-tadalafil, macitentan-sildenafil, and ambrisentan-tadalafil)^6^, ^23^, ^24^ and the fourth therapy was modeled off a STCT of macitentan-tadalafil.^25^ Each regimen displayed information on its dosing, discontinuation rates due to side effects, titration, number of pharmacies needed to visit, and number of prior authorizations required; these factors were selected from a panel of PAH practitioners as being relevant to treatment selection and important in determining the utility of STCT in this disease area. Due to individual differences in insurance coverage, an accurate out-of-pocket cost for each regimen could not be reliably presented; thus, we omitted cost as a feature in the blinded choice task. Separate questionnaire items further assessed respondents' attitudes and beliefs about STCT.

### Sample size

A general rule for estimating sample sizes for a traditional choice-based conjoint analysis is $\frac{\mathrm{nta}}{c}\geq 500.$^26^, ^27^ In this formula, *n* = number of respondents, *t* = number of choice tasks, *a* = number of alternatives (profiles) per task, and *c* = largest number of levels for any one attribute. Our design yields a formula result of 1,206 with a sample size of 201, indicating sufficient sample size to generate relatively precise preference weights. We further implemented an adaptive choice-based conjoint design as it collects more data per participant than a traditional DCE and allows for greater stabilization of estimates in designs with smaller sample sizes.^28^

### Statistical analysis

Data were analyzed using SAS Studio 3.81 (for descriptive statistics) and Sawtooth Software Lighthouse Studio 9.15.9 (for the DCE analysis). Preference weights were generated for each attribute level via hierarchical Bayesian estimation.^29^ These preference weights allow one to understand preferences *within* each attribute (i.e., how one attribute level is valued relative to its alternatives): higher weights indicate greater preference (or odds of being selected) whereas lower weights indicate lower preference or aversion. Relative importance estimates were also generated for each attribute by calculating the difference between the highest and lowest preference weights for each attribute (i.e., the most preferred vs least preferred levels), dividing by the sum of all attributes’ ranges, and multiplying by 100 to convert into a percentage. In contrast with preference weights, which are used for understanding within-attribute patterns, relative importance scores enable one to directly compare between attributes—with the largest values reflecting the most important factors driving patient decision-making. Descriptive statistics were used to summarize the aggregate sample (N = 201). Frequency counts and percentages were provided for categorical variables, whereas means and standard deviations were provided for continuous or discrete variables.

## Results

### Participant characteristics

Respondents were predominantly White (86.1%) and female (88.6%). The majority were not employed (56.2%), with over one-third reporting a disability (38.3%) and one-third reporting a World Health Organization Functional Class (WHO FC) III-IV disease (31.4%). Most had health insurance: 71.1% were covered by public insurance (of which 55.7% were Medicare), and 56.2% were covered by private insurance (Table 1).

**Table 1:** Patient Characteristics

	All respondents (N = 201)	
	N/Mean	%/SD
Age		
Mean (SD)	57.6	13.9
Median (range)	59.0	21-85
Sex, *n* (%)		
Male	23	11.4%
Female	178	88.6%
Prefer not to answer	0	
Ethnicity, *n* (%)		
Non-Hispanic	183	91.0%
Hispanic	16	8.0%
Prefer not to answer	2	1.0%
Race, *n* (%)^a^		
White	173	86.1%
Black or African American	10	5.0%
American Indian or Alaska Native	0	
Asian	6	3.0%
Native Hawaiian or other Pacific Islander	1	0.5%
Middle Eastern or North African	0	
Two or more races	5	2.5%
Prefer not to answer	6	3.0%
US region, *n* (%)		
West	56	27.9%
Midwest	46	22.9%
Northeast	27	13.4%
South	72	35.8%
Education, *n* (%)		
Some high school, no diploma	3	1.5%
High school graduate or equivalent	20	10.0%
Some college, no degree	45	22.4%
Associate’s degree	30	14.9%
Bachelor’s degree	66	32.8%
Post-bachelor’s degree	37	18.4%
Prefer not to answer	0	
Employment, *n* (%)		
Employed full-time	35	17.4%
Self-employed	11	5.5%
Employed part-time	14	7.0%
Manage family/household	21	10.5%
Not employed and looking for work	3	1.5%
Not employed and not looking for work (e.g., student, retired)	33	16.4%
Not employed and unable to work (e.g., disability)	77	38.3%
Prefer not to answer	7	3.5%
Insurance, n (%) *Select all that apply*		
Public Insurance	143	71.1%
Medicare	112	55.7%
Medicaid	28	13.9%
VA	3	1.5%
Private Insurance	113	56.2%
Other insurance	9	4.5%
Access to support systems, *n* (%) *Select all that apply*		
Professional care provider	87	43.3%
Family caregiver	64	31.8%
Patient organization assistance program	63	31.3%
Patient support program	61	30.4%
Vouchers and/or discount cards that reduce the cost of medication	37	18.4%
Other	33	16.4%
Government disability assistance	24	11.9%
Caregiver, *n* (%) *Select all that apply*		
Spouse/partner	118	58.7%
Professional care provider	46	22.9%
Child(ren)	33	16.4%
Other	29	14.4%
Extended family	28	13.9%
Neighbor	4	2.0%

Abbreviation: SD, standard deviation; VA, veteran's affairs.

a Race recoded to exclusive categories.

Over one-third of respondents had access to support systems, including PAH professional care providers (43.3%), family caregivers (31.8%), and/or received support from patient assistance/support programs (31.3%-30.4%). Over half received caregiving support from their spouse/partner (58.7%). Vouchers and/or discount cards were used by 18.4% to reduce the cost of their medication.

On average, patients had been on their current PAH medication for 7.5 ± 6.1 years, which was typically double (17.9%) or triple (36.8%) therapy containing an ERA + PDE5i; the remainder were using monotherapy of ERA or PDE5i (26.4%) or soluble guanylate cyclase stimulators-based combination therapies (11%). Mean out-of-pocket costs averaged $96 ± 203 per month, with many individuals paying <$20 per month (median, range = $15, $0-1,100) **(**Table 2).

**Table 2:** Patient Health and Treatment Characteristics

	All respondents (N = 201)	
	N/Mean	%/SD
Health conditions, *n* (%)		
Pulmonary arterial hypertension (WHO group 1)	201	100.0%
Sleep apnea	79	39.3%
Obesity	49	24.4%
Depression	44	21.9%
Thyroid disease	39	19.4%
Anemia	28	13.9%
Diabetes	20	10.0%
Chronic kidney disease	19	9.5%
Systemic hypertension	16	8.0%
Cancer	15	7.5%
Chronic pain	13	6.5%
Chronic liver disease	4	2.0%
Peripheral vascular disease	1	0.5%
Current treatment, *n* (%)		
PDE5i (sildenafil or tadalafil) + ERA (bosentan, ambristan, or macitentan) + prostacyclin (selexipag, treprostinil, epoprostenol, or iloprost)	74	36.8%
PDE5i (sildenafil or tadalafil) + ERA (bosentan, ambristan, or macitentan)	36	17.9%
PDE5i alone (sildenafil or tadalafil)	31	15.4%
ERA alone (bosentan, ambrisentan, or macitentan)	22	11.0%
Other	16	8.0%
sGC (riociguat) + ERA (bosentan, ambrisentan, or macitentan) + prostacyclin (selexipag, treprostinil, epoprostenol, or iloprost)	15	7.5%
sGC (riociguat) + ERA (bosentan, ambrisentan, or macitentan)	7	3.5%
Current medication regimen duration, years		
Mean (SD)	7.5	6.1
Median (range)	6.3	0.25-37.1
Time since diagnosis, years^a^		
Mean (SD)	10.6	8
Median (range)	9	1-38
Time with provider, years		
Mean (SD)	7.0	5.7
Median (range)	6	0.08-28.8
WHO functional class symptoms, *n* (%)		
Class I	42	20.9%
Class II	96	47.7%
Class III	58	28.9%
Class IV	5	2.5%
Current regimen out-of-pocket cost, $^b^		
Mean (SD)	96	203.1
Median (range)	15	0-1100

Abbreviations: ERA, endothelin receptor antagonist; PDE5i, phosphodiesterase-5 inhibitor; SD, standard deviation; sGC, soluble guanylate cyclase stimulators; WHO, World Health Organization.

a An outlier reporting 106 years in the “Time Since Diagnosis” category was removed for inconsistencies with “Current Med Duration” and “Time with Provider.”

b Two outliers that reported exceptionally high costs ($5,000 and $7,000) were removed as being unlikely.

### Treatment factors influencing ERA + PDE5i adoption

**Figure 1:**
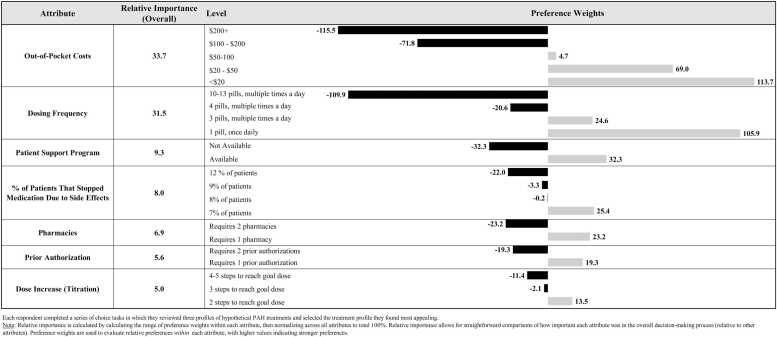
Each respondent completed a series of choice tasks in which they reviewed 3 profiles of hypothetical PAH treatments and selected the treatment profile they found most appealing. Note: Relative importance is calculated by calculating the range of preference weights within each attribute, then normalizing across all attributes to total 100%. Relative importance allows for straightforward comparisons of how important each attribute was in the overall decision-making process (relative to other attributes). Preference weights are used to evaluate relative preferences within each attribute, with higher values indicating stronger preferences. PAH, pulmonary arterial hypertension.

Relative importance estimates (S1.1) indicated that the most important factors (of those tested) influencing ERA + PDE5i adoption were lower out-of-pocket costs (33.7) and a simpler dosing regimen (31.5). Other factors such as patient support program (available vs not available = 9.3), discontinuation due to side effects (8.0), number of pharmacies required to obtain medications (1 vs 2 = 6.9), number of prior authorizations required (1 vs 2 = 5.6), and dose increase (titration) (5.0) were less important in determining treatment choices (Figure 1).

**Figure 2:**
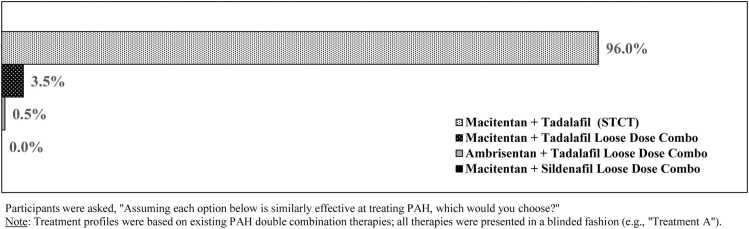
Participants were asked, “Assuming each option below is similarly effective at treating PAH, which would you choose?” Note: Treatment profiles were based on existing PAH double combination therapies; all therapies were presented in a blinded fashion (e.g., “Treatment A”). PAH, pulmonary arterial hypertension; STCT, single tablet combination therapy.

Individual preference weights (Figure 2) revealed that patients were *most* likely to select ERA + PDE5i treatment when out-of-pocket costs were under $20 (113.7), dosing regimen was 1 pill, once daily (105.9), patient support programs were available (32.3), discontinuation due to side effects was the least likely at 7% (25.4), the medications required 1 pharmacy (23.2), required 1 prior authorization (19.3), and only 2 steps were needed to reach goal dose (13.5) compared to 3 or 4 and 5 steps.

Further exploration confirmed that preferences for the lowest dosing regimen (single tablet therapy) and the lowest out-of-pocket cost (<$20) were maintained, despite patients’ current therapy (monotherapy vs double/triple therapy) or presumed financial status (inferred by their use of public only vs private/mixed insurance) (S1.3-S1.4).

### Blinded choice of PAH therapies

**Figure 3:**
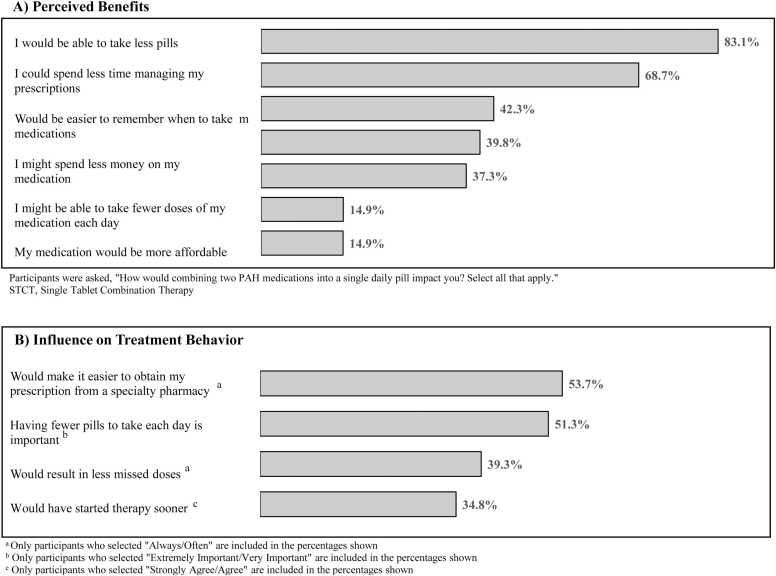
(A) Participants were asked, “How would combining two PAH medications into a single daily pill impact you? Select all that apply.” (B) (a) Only participants who selected “Always/Often” are included in the percentages shown. (b) Only participants who selected “Extremely Important/Very Important” are included in the percentages shown. (c) Only participants who selected “Strongly Agree/Agree” are included in the percentages shown. STCT, single tablet combination therapy.

When asked to select among blinded regimens mimicking the real-world characteristics of loose-dose ERA + PDE5i therapies, in addition to a hypothetical STCT, most respondents selected a profile resembling a STCT of macitentan-tadalafil (96.0%) (Figure 3).

### Perceptions of STCT of ERA + PDE5i

Most respondents noted that STCT would allow them to take fewer pills per day (83.1%) and reduce time spent managing prescriptions (68.7%). Over one-third identified benefits to adherence (42.3%), cost (39.8%), and reduced pill burden (37.3%). Few expected their medications to be more affordable (14.9%) or associated STCT with increased treatment quality or confidence in physician-recommended medications (14.9%) (Figure 3A).

Likert scales indicated mixed evidence for whether an STCT would prove useful for compliance (Always/Often = 39.3%; Rarely/Never = 39.3%) and/or initiation of combination therapy (Strongly Agree/Agree = 34.8%; Disagree/Strongly Disagree = 30.8%). However, preferences for reduced pill burden (Always/Often = 51.3%) and easier prescription access (Always/Often = 53.7%) were upheld, consistent with the DCE and other self-report items (Figure 3B). The complete results can be found in the Appendix (S1.2a-c).

## Discussion

This study examined the factors influencing patients with PAH’s decision-making regarding PDE5i + ERA therapy and their interest in STCT. Results of the DCE showed that patients were most receptive to combination therapies when offered with the lowest cost and least frequent dosing (i.e., lower pill burden). Cost slightly outweighed the convenience of single tablet dosing, aligning with findings from other chronic diseases such as diabetes and hypertension, where high out-of-pocket expenses reduce adherence.^30^ Participants were willing to trade off other benefits to reduce pill burden, which was 3 times as important as factors such as avoiding side effects or accessing support programs. Given that many participants had long-standing PAH and were already on combination therapy, concerns about side effects were minimal and factors such as titration and prior authorizations played a minor role in decision-making.

Most participants saw STCT as a promising solution for improving adherence by simplifying treatment regimens—a factor that is especially important in a population observed to take as many as 12 pills per day.^12^ This result is consistent with prior studies in other disease states showing that complex medication regimens contribute to reduced adherence and increased burden,^33^, ^34^, ^35^, ^39^ whereas simplified regimens—including STCT regimens^12^, ^13^, ^14^, ^15^, ^16^, ^17^, ^18^, ^19^—can lead to better disease control^13^, ^14^, ^15^, ^16^, ^17^, ^18^, ^19^ and lower health care resource utilization.^36^, ^39^ Our participants recognized this potential benefit, showing a strong preference for STCT in the DCE (when hypothetical treatment profiles were presented), as well as in the blinded choice task (when real-world treatment profiles were presented). In the latter case, 96% preferred a treatment simulating a STCT over multipill, “loose dose” alternatives (<4%).

Medication cost was a decisive factor, regardless of insurance type, with both publicly and privately insured patients favoring lower out-of-pocket expenses (S1.3). This is not surprising as most participants in this study had financial challenges such as unemployment and reliance on public insurance, which likely shaped their treatment preferences. Interestingly, while some participants additionally reported using vouchers or manufacturer support programs to manage costs, these programs were found largely unimportant to determining patient decision-making in the DCE. These findings highlight the need for clinicians to incorporate cost discussions into shared decision-making and to increase awareness of support programs that can help bridge financial barriers.

Although participants in this study strongly preferred simplified regimens such as STCT, they also prioritized minimizing out-of-pocket costs—2 preferences that are often at odds with one another. STCT typically comes at a higher price point due to their formulation complexity and branded status, presenting a financial barrier for patients who are already economically vulnerable. To reconcile this tension, strategies are needed to make STCT more affordable and accessible. These include expanded eligibility and awareness of manufacturer-sponsored copay and voucher programs, greater insurer coverage of STCT on preferred drug formularies, and value-based pricing models that account for long-term cost savings from improved adherence and reduced health care utilization. Additionally, policy efforts aimed at reducing cost-sharing burdens—particularly for publicly insured individuals—could help ensure that financial constraints do not prevent patients from choosing the therapies most likely to support sustained treatment success.

## Limitations

The results of this study must be considered within the context of its limitations. Participants were a convenience sample of English-speaking US patients enrolled in the PHAR registry, which may limit generalizability to nonregistry populations and non-US settings. The sample was skewed toward White, insured women, and thus may not reflect the treatment priorities of uninsured and/or underinsured patients, especially those of minority groups that disproportionately experience issues with health care affordability and insurance access.^37^, ^38^ Over half of participants were not employed at the time of study, and this may reflect a sampling bias wherein unemployed adults are more likely to participate in online surveys. As a prevalent population, these patients had lived with their disease for some time, likely adjusting to their condition, which could result in different responses compared to those at the start of their treatment journey. While these sampling limitations may result in certain patients being omitted from the survey, the results nevertheless reflect valid preferences collected among WHO group 1 patients with PAH residing in the United States.

As with any DCE, the study results are bound by the attributes and levels included. To minimize respondent burden, fewer than 10 attributes were tested, and some important factors—such as clinical outcomes—were omitted. While the results observed here are valid within the choices provided to respondents, it is possible that decision-making would differ had respondents been shown a different selection of attributes and levels. Likewise, the blinded choice task—which omitted the cost of each regimen but included features such as dosing, discontinuation rates, and prior authorizations—may have yielded different results had a different combination of regimen characteristics been presented. Lastly, there is always some risk of inaccuracy in self-reported surveys; however, the structured nature of the DCE reduces the opportunity for distortions in memory by eliminating the need for open-ended recall, and the remaining questionnaire items represent low-stakes situations that are unlikely to evoke bad-faith answers.

## Conclusions

Uptake of guideline–recommended upfront double therapy remains low in both the United States and globally, with monotherapy still commonly used in practice.^8^, ^32^ The results of this study suggest that dosing regimen (i.e., pill burden) is a critical factor influencing treatment selection among adults with PAH, and that STCT represents a way for patients to simplify their regimens and improve adherence. Whether STCT is effective in increasing adoption of guideline-recommended therapy and optimizing patient outcomes will ultimately depend on its affordability, as many patients face financial limitations and are strongly motivated to select the lowest-cost therapeutic option. While the upfront costs of combination therapy (including STCT) are likely to be offset in the long-term as a function of better disease management,^3^, ^31^ near-term solutions for reducing out-of-pocket costs are needed to ensure STCT remains an option available to patients.

## Author contributions

**Gabriela Gomez Rendon:** Conceptualization, Methodology, Writing - review and editing. **David Lopez:** Conceptualization, Methodology, Writing - review and editing. **Carly J. Paoli:** Conceptualization, Methodology, Writing - review and editing. **Mohammad Rahman:** Methodology, Writing - review and editing. **Abraham Lee:** Data curation, Software, Formal analysis; **November McGarvey:** Conceptualization, Methodology, Supervision. **Sana Mirza:** Data curation, Formal analysis, Writing – original draft. **Ashley Martin:** Conceptualization, Methodology, Formal analysis, Writing – original draft. **Nicholas A. Kolaitis:** Conceptualization, Methodology, Writing - review and editing. **Lana Melendres-Groves:** Conceptualization, Methodology, Writing - review and editing. **Melisa Wilson:** Conceptualization, Methodology, Writing - review and editing. **Martha Kingman:** Conceptualization, Methodology, Writing - review and editing.

## Funding

This study was sponsored by Johnson & Johnson Innovative Medicine, Titusville, NJ.

## Declaration of Competing Interest

Nicholas A. Kolaitis reports administrative support, article publishing charges, statistical analysis, and writing assistance were provided by Janssen Pharmaceuticals Inc. Gabriela Gomez Rendon reports a relationship with Johnson & Johnson Innovative Medicine that includes employment and equity or stocks. David Lopez reports a relationship with Johnson & Johnson Innovative Medicine that includes employment and equity or stocks. Martha Kingman reports a relationship with Johnson & Johnson Innovative Medicine that includes consulting or advisory. Carly J. Paoli reports a relationship with Janssen Pharmaceuticals Inc that includes employment and equity or stocks. Mohammad Rahman reports a relationship with Janssen Pharmaceuticals Inc that includes employment and equity or stocks. Ashley Martin reports a relationship with BluePath Solutions Inc that includes employment. November McGarvey reports a relationship with BluePath Solutions Inc that includes employment. Abraham Lee reports a relationship with BluePath Solutions Inc that includes employment. Sana Mirza reports a relationship with BluePath Solutions Inc that includes employment. Nicholas Kolaitis reports a relationship with Johnson & Johnson Innovative Medicine that includes consulting or advisory. Johnson & Johnson Innovative Medicine reports a relationship with Melisa Wilson that includes consulting or advisory. Lana Melendez-Groves reports a relationship with Johnson & Johnson Innovative Medicine that includes consulting or advisory. G. Gomez, D. Lopez, C.J. Paoli, M. Rahman: Employees of Johnson & Johnson Innovative Medicine and hold stock in J&J. A. Lee, and N. McGarvey: Were employees of BluePath Solutions (BPS) at time of research but are no longer employed by BPS. N.A. Kolaitis: Has received financial support from J&J, Liquidia, United Therapeutics, Bayer. M. Wilson: Has received financial support from J&J, Merck, Bayer, United Therapeutics. M. Kingman: Has received financial support from Aerami, Merck, J&J, Liquidia, Gossamer. L. Melendres: Has received financial support from J&J, United Therapeutic, Bayer, Merck. The other authors declare that they have no known competing financial interests or personal relationships that could have appeared to influence the work reported in this paper.

## Footnotes

Appendix A Supplementary data associated with this article can be found in the online version at doi:10.1016/j.jhlto.2025.100410.
