# Supplementary material for: Exploring patient preferences regarding the use of combination therapy with endothelin receptor antagonist (ERA) + phosphodiesterase-5 inhibitors (PDE5i)
Source: JHLT Open. 2025 Oct 15;11:100410. doi: 10.1016/j.jhlto.2025.100410 (PMC12639287; doi:10.1016/j.jhlto.2025.100410)
Supplement: Supplementary file 1 — Supplementary material [file mmc1.pdf]

SUPPLEMENT

S0. Study Material

S0.1 Example Choice Trial

Please indicate whether each PAH treatment below is a possibility or not for you. ***Please assume that all treatments are similarly effective at treating PAH. ***

Treatment Characteristics	Treatment A	Treatment B	Treatment C
**Out-of-Pocket Costs**	<$25	$50-100	$50-100
**Dosing**	One pill, once daily	Three pills, multiple times a day	Ten to thirteen pills, multiple times a day
**Prior Authorizations**	Requires 1 prior authorization	Requires 2 prior authorizations	Requires 1 prior authorization
**Pharmacies**	Requires 1 pharmacy	Requires 1 pharmacy	Requires 2 pharmacies
**Discontinuation Due to Side Effects**	8%	7%	9%
**Dose Increase (Titration)**	Available	Not available	Available
**Patient Support Program**	Available	Available	Not available
	○ A possibility<br>○ Not a possibility	○ A possibility<br>○ Not a possibility	○ A possibility<br>○ Not a possibility

S0.2. Attributes for Patient Survey

The following attributes were identified from literature review and agreed upon by a panel of clinical experts as being relevant to the PAH decision-making process.

Treatment Characteristic	Response Options/Levels
**Out-of-Pocket Costs**	• <$20<br>• $20-50<br>• $50-100<br>• $100-200<br>• $200+
**Dosing**	• One pill, once daily<br>• Three pills, multiple times a day<br>• Four pills, multiple times a day<br>• Ten to thirteen pills, multiple times a day
**% Patients that stopped medication due to side effects**	• 7% chance<br>• 8% chance<br>• 9% chance<br>• 12% chance
**Prior Authorizations**	• Requires 1 prior authorization<br>• Requires 2 prior authorizations
**Pharmacies**	• Requires 1 pharmacy<br>• Requires 2 pharmacies
**Dose Increase (Titration)**	• 2 steps to reach goal dose<br>• 3 steps to reach goal dose<br>• 4-5 steps to reach goal dose
**Patient Support Program**	• Available<br>• Not Available

S0.3 Blinded Choice Exercise

Treatment Characteristics	Treatment A	Treatment B	Treatment C	Treatment D
***Blinded* Therapy Profile**	*Macitentan + Tadalafil Single Tablet Combination Therapy*	*Macitentan + Tadalafil Loose Dose*	*Macitentan + Sildenafil** Loose Dose*	*Ambrisentan + Tadalafil Loose Dose*
**Dosing regimen**	1 tablet once daily	3 tablets per day	Up to 10-13 tablets per day	Up to 3 tablets per day
**Dose Increase (Titration)**	Available	Available	Available	Available
**Discontinuation Due to Side Effects***	8%	7%	9%	12%
**Pharmacies Required**	Requires 1 pharmacy	Requires 2 pharmacies	Requires 2 pharmacies	Requires 2 pharmacies
**Prior authorization**	Requires 1 prior authorization	Requires 2 prior authorizations	Requires 2 prior authorizations	Requires 2 prior authorizations

Note: Treatment profiles were be blinded (unbranded). Safety/tolerability and efficacy data were obtained from the A DUE study (Grunig et al., 2024)), OPTIMA (Sitbon et al., 2020), SERAPHIN (Janse & Pulido, 2017), and AMBITION (Galie et al., 2015) clinical trials; SERAPHIN data are reported from a subset of N=154 on background therapy, of whom 150 were taking PDE5i. As a panel of clinical experts advised that all treatments are viewed as similarly efficacious, we omitted specific efficacy data (e.g., 6MWD, PVR) from the exercise but pointed out their similar efficacy in the question wording; this was done to ensure respondents had comprehensive insight into the relative effectiveness of these blinded treatment profiles. ^Assumes max dose of 60-80mg sildenafil TID.

* Presented to respondents in the DCE as "the percentage of patients that stopped medication due to side effects"

S1. Study Results

S1.1 Relative Importance of Each Attribute (Overall)

Attribute	All Respondents (N=201)			
			95% CI	
	Mean	SD	Lower	Upper
**Dose Increase (Titration)**	5.0	2.8	4.6	5.3
**Prior Authorization**	5.6	3.4	5.2	6.1
**Pharmacies**	6.9	4.6	6.3	7.6
**Discontinuation Due to Side Effects***	8.0	3.9	7.4	8.5
**Patient Support Program**	9.3	8.2	8.2	10.4
**Dosing regimen**	31.5	11.9	29.9	33.2
**Out-of-Pocket Costs**	33.7	13.9	31.7	35.6

Note: Relative importance is calculated by dividing the range of each attribute (the utility of the highest level minus the utility of the lowest level) by the sum of ranges of all attributes and multiplying by 100. The sum of all relative importance scores is 100% and an attribute with 20% relative importance is interpreted as being twice as important as an attribute with 10% relative importance. Thus, the estimates indicate how important each attribute is when deciding to choose a treatment.

* Presented to respondents in the DCE as "the percentage of patients that stopped medication due to side effects"

S1.2a Influence of STCT on Treatment Behavior

**Beliefs Regarding STCT**	**All Respondents (N=201)**	
	**N**	**%**
**Compliance, n (%)**<br>*Would combining two PAH medications into a single daily pill result in less missed doses of your medication?*		
Always	50	24.9%
Often	29	14.4%
Sometimes	43	21.4%
Rarely	35	17.4%
Never	44	21.9%
**Access, n (%)**<br>*Would combining two PAH medications into a single daily pill make it easier to obtain your prescription from a specialty pharmacy?*		
Always	74	36.8%
Often	34	16.9%
Sometimes	45	22.4%
Rarely	19	9.5%
Never	29	14.4%
**Pill Burden, n (%)**<br>*How important is it to you to have less pills to take every day?*		
Extremely important	61	30.4%
Very important	42	20.9%
Somewhat important	66	32.8%
Not at all important	32	15.9%
**Initiation, n (%)**<br>*If you could combine two PAH medications into a single daily pill, do you feel you would you have started combination therapy sooner?*		
Strongly Agree	40	19.9%
Agree	30	14.9%
No opinion/unsure	69	34.3%
Disagree	31	15.4%
Strongly disagree	31	15.4%

S1.2b Perceived Benefits of STCT

Pros of STCT	All Respondents (N=201)	
	**N**	**%**
How would combining two PAH medications into a single daily pill impact you? Select all that apply.		
I would be able to take less pills	167	83.1%
I could spend less time managing my prescriptions (for example: requesting refills, completing paperwork, driving to the pharmacy)	138	68.7%
I would find it easier to remember when to take my medication and stick to my medication schedule	85	42.3%
I might spend less money on my medication	80	39.8%
I might be able to take fewer doses of my medication each day	75	37.3%
My medication would be more affordable	30	14.9%
I would feel more confident in my medication because it is recommended by my physician or the medical guidelines	30	14.9%
None of the above	11	5.5%

S1.2c General Beliefs about Medication

General Beliefs About Medications, n (%)	All Respondents (N=201)	
	N	%
I believe that side effects naturally lessen / get better over time		
Strongly Agree	28	13.9%
Agree	88	43.8%
No opinion/unsure	40	19.9%
Disagree	43	21.4%
Strongly disagree	2	1.0%
I believe that side effects can be managed		
Strongly Agree	28	13.9%
Agree	118	58.7%
No opinion/unsure	32	15.9%
Disagree	22	11.0%
Strongly disagree	1	0.5%
I believe it is OK to miss doses of my medication sometimes		
Strongly Agree	0	
Agree	7	3.5%
No opinion/unsure	17	8.5%
Disagree	79	39.3%
Strongly disagree	98	48.8%
I believe medications always have side effects		
Strongly Agree	23	11.4%
Agree	95	47.3%
No opinion/unsure	29	14.4%
Disagree	48	23.9%
Strongly disagree	6	3.0%
I believe medications should have an immediate effect		
Strongly Agree	8	4.0%
Agree	50	24.9%
No opinion/unsure	66	32.8%
Disagree	74	36.8%
Strongly disagree	3	1.5%
I am willing to slowly increase my medication to lessen side effects		
Strongly Agree	67	33.3%
Agree	113	56.2%
No opinion/unsure	16	8.0%
Disagree	4	2.0%
Strongly disagree	1	0.5%

S1.3 Relative Importance of Each Attribute – Insurance (Public vs. Private)

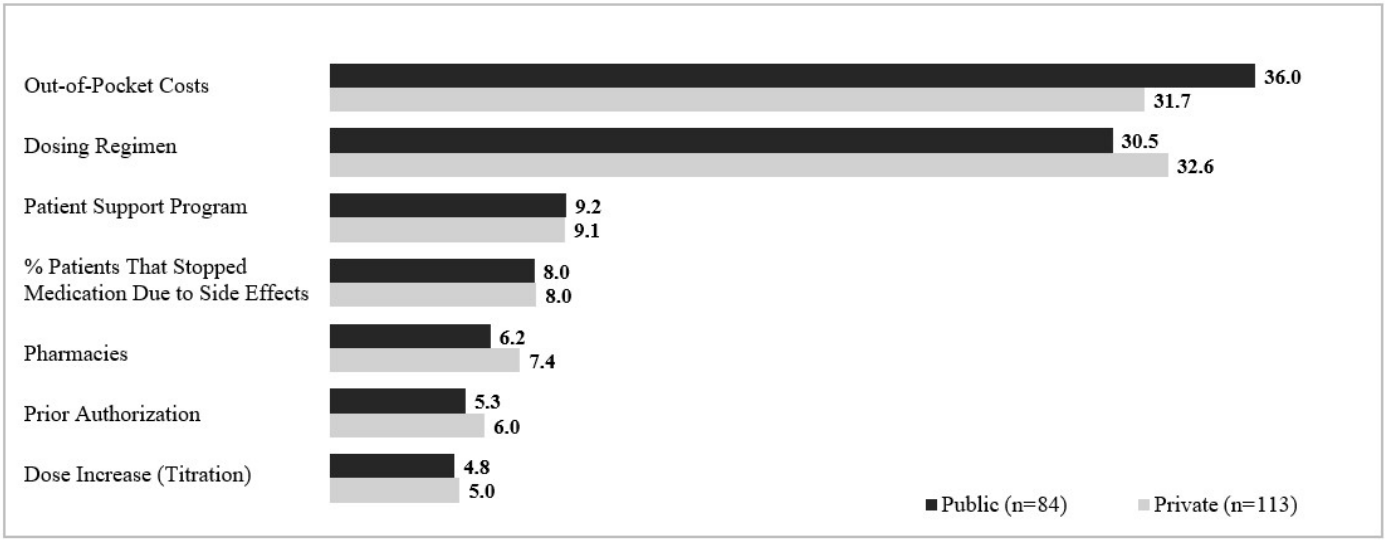

S1.4 Relative Importance of Each Attribute – Therapy (Combination vs. Monotherapy)

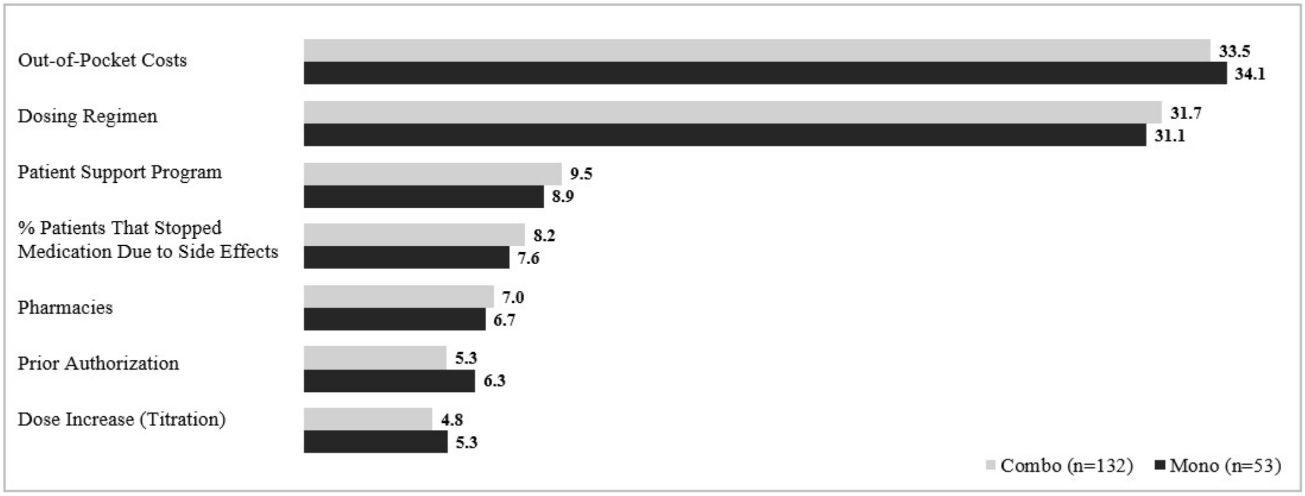

DIRECT Checklist for reporting discrete choice experiments in health

**Section** **Item**		**Page and paragraph**
**Purpose and rationale**		
**1**	Describe the real-world context and decision-maker that the hypothetical choice context seeks to replicate or inform	pg. 5; paragraph 1
**2**	Provide a rationale for using a DCE to answer the research question	pg. 6; paragraph 2
**Attributes and levels**[a]		
**3**	Describe how attributes and levels were derived (e.g. literature review, interviews, focus groups, expert input)	pg. 7; paragraph 1
**4**	Provide the final list of attributes and levels	pg. 6; paragraph 3
**Experimental design**		
**5**	Report the number of alternatives per choice set and whether they were labelled or unlabelled	pg. 6; paragraph 3
**6**	Describe response options (e.g. forced choice, opt-out, status quo)	pg. 6; paragraph 3
**7**	Describe the type of experimental design (e.g. orthogonal, D-efficient, Bayesian efficient, partial profile)	pg. 7; paragraph 3
**8**	Describe which effects are identified in the design (e.g. main effects, higher order interactions, functional form)	pg. 7; paragraph 3
**9**	Describe the number of choice sets, blocks and choice sets per block	
**10**	Indicate how the experimental design was obtained (software, catalogue, other)	pg. 8; paragraph 4
**Survey design**		
**11**	Provide a sample choice set and the instructions and background information given to respondents (e.g. providing the survey as an appendix)	pg. 6; paragraph 3

**12**	Report any randomisation (e.g. choice set order, attribute order, alternative order, framing effects)	pg. 6; paragraph 3
**13**	Describe what was checked in piloting (e.g. understanding, respondent burden, timing, wording)	pg. 5; paragraph 2
**14**	Report whether information from the pilot was used to update the experimental design (e.g. priors, functional form of attributes) or survey design	pg. 5; paragraph 2
**Sample and data collection**		
**15**	Report respondent inclusion/exclusion criteria	pg. 5; paragraph 3
**16**	Describe how data were collected (e.g. mail, personal interview, web survey)	pg. 5; paragraph 3
**17**	Report the response rate or cooperation rate, if possible	n/a
**18**	Report the final sample size and how the sample size was determined	pg. 5; paragraph 3
**19**	Describe respondent characteristics and representativeness of target population, if known	pg. 9; paragraph 1
**Econometric analysis**		
**20**	Indicate coding of data (e.g. effects, dummy, continuous) including definitions	n/a
**21**	Report whether any respondents were removed and why (e.g. suspected fraudulent responses, rationality tests)	n/a
**22**	Provide the rationale for model choice (e.g. conditional logit, mixed logit, latent class) and assumptions (e.g. error variance)	pg. 8; paragraph 1
**23**	Report model specification	pg. 8; paragraph 1
**Reporting of results**		
**24**	Report the model performance, goodness of fit (if comparing models)	n/a
**25**	Describe methods used for analysis of model results (e.g. calculation of marginal rate of substitution, attribute relative importance, welfare gain)	pg. 8; paragraph 3

**26**	Report measures of precision for the output(s) of interest (e.g. confidence intervals) and how these were derived	pg. 8; paragraph 2
